# Supplementary material for: NLRP3 and AIM2 inflammasomes exacerbate the pathogenic Th17 cell response to eggs of the helminth Schistosoma mansoni
Source: bioRxiv. 2024 Mar 13:2024.03.11.584371. Preprint. [Version 1] doi: 10.1101/2024.03.11.584371 (PMC10979858; doi:10.1101/2024.03.11.584371)
Supplement: 1 [file NIHPP2024.03.11.584371V1-supplement-1.pdf]

Supporting Information

bioRxiv preprint doi: https://doi.org/10.1101/2024.03.11.584371; this version posted March 13, 2024. The copyright holder for this preprint (which was not certified by peer review) is the author/funder, who has granted bioRxiv a license to display the preprint in perpetuity. It is made available under aCC-BY 4.0 International license.

S Fig 1 (related to Fig 3)

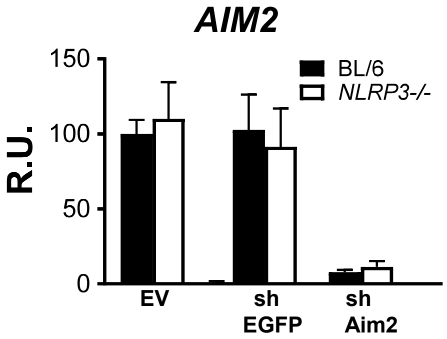

S Fig 1 (related to Fig 3): Verification of AIM2 knockdown in BMDCs. BL/6 and *NLRP3*$^{-/-}$ BMDCs were transduced with empty vector (EV), EGFP shRNA (shEGFP) or AIM2 shRNA (shAIM2). The mRNA levels of AIM2 were set at 100% in cells transduced with EV. AIM2 mRNA levels were assessed by qRT-PCR. Bars represent the mean ±S.D. AIM2 relative units (R.U.) of three biological replicates from one representative experiment of three with similar results.

bioRxiv preprint doi: https://doi.org/10.1101/2024.03.11.584371; this version posted March 13, 2024. The copyright holder for this preprint (which was not certified by peer review) is the author/funder, who has granted bioRxiv a license to display the preprint in perpetuity. It is made available under aCC-BY 4.0 International license.

S Fig 2 (related to Fig 4)

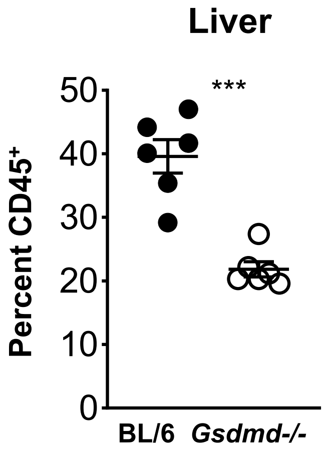

S Fig 2 (related to Fig 4): Total CD45+ populations were decreased in the *Gsdmd*-/- livers. Total CD45+ populations in liver cells isolated from C57BL/6 and Gsdmd-/- mice infected with *Schistosoma mansoni* for 7 weeks. Data are representative of two independent experiments. Significance was determined using a Student's t-test ***$p < 0.0005$.

bioRxiv preprint doi: https://doi.org/10.1101/2024.03.11.584371; this version posted March 13, 2024. The copyright holder for this preprint (which was not certified by peer review) is the author/funder, who has granted bioRxiv a license to display the preprint in perpetuity. It is made available under aCC-BY 4.0 International license.
